# Economics of autonomous equipment for arable farms

**DOI:** 10.1007/s11119-021-09822-x

**Published:** 2021-05-27

**Authors:** James Lowenberg-DeBoer, Kit Franklin, Karl Behrendt, Richard Godwin

**Affiliations:** Harper Adams University, Shrophire, Newport, TF10 8NB UK

**Keywords:** Autonomous equipment, Robots, Economic feasibility, Economy of size, Grain production

## Abstract

By collecting more data at a higher resolution and by creating the capacity to implement detailed crop management, autonomous crop equipment has the potential to revolutionise precision agriculture (PA), but unless farmers find autonomous equipment profitable it is unlikely to be widely adopted. The objective of this study was to identify the potential economic implications of autonomous crop equipment for arable agriculture using a grain-oilseed farm in the United Kingdom as an example. The study is possible because the Hands Free Hectare (HFH) demonstration project at Harper Adams University has produced grain with autonomous equipment since 2017. That practical experience showed the technical feasibility of autonomous grain production and provides parameters for farm-level linear programming (LP) to estimate farm management opportunities when autonomous equipment is available. The study shows that arable crop production with autonomous equipment is technically and economically feasible, allowing medium size farms to approach minimum per unit production cost levels. The ability to achieve minimum production costs at relatively modest farm size means that the pressure to “get big or get out” will diminish. Costs of production that are internationally competitive will mean reduced need for government subsidies and greater independence for farmers. The ability of autonomous equipment to achieve minimum production costs even on small, irregularly shaped fields will improve environmental performance of crop agriculture by reducing pressure to remove hedges, fell infield trees and enlarge fields.

**Supplementary Information:**

The online version contains supplementary material available at 10.1007/s11119-021-09822-x.

## Introduction

The vision of the farm of the future has long assumed that robots and automation would play a major role (Asseng & Asche, 2019; Holt, 1985; Morgan, 1961). Researchers, academics and business leaders expect robots and autonomous equipment to substantially increase the capacity of farmers for precision agriculture (PA) in the form of increased spatial and temporal management of crops and livestock (see for example, Robotics Business Review, 2016; Shamshiri et al., 2018; Duckett et al., 2018). Interest in farm automation and robotics has been renewed by the farm labour shortages in Europe and North America during the COVID-19 pandemic in 2020 (e.g. Charlton and Castillo 2021). Economic feasibility is key to achieving the social and environmental benefits of robotic and autonomous agriculture such as: reduction in human drudgery; alternatives to chemical pest control, reducing soil compaction and farming small irregularly shaped fields (Duckett et al., 2018). Conventional mechanization has been very successful in agricultural topographies with relatively large rectangular fields, but it has been less successful where fields are small and irregularly shaped. In some countries with mechanized agriculture, those small, irregularly shaped fields have been largely abandoned (e.g. USA) and, in others, production has been subsidized (e.g. European Union and Switzerland). Rigorous economic analyses of the economic feasibility of farms using robotic and autonomous equipment are rare primarily because it is early days for this technology. Most public sector research on crop robotics is at most in the prototype stage without enough field experience to make credible economic estimates. Private sector crop robots are proprietary technology and little information is released. This economic analysis is made possible through the experience of the Hands Free Hectare (HFH–http://www.handfreehectare.com) demonstration project at Harper Adams University, Newport, UK, which showed that small to medium scale conventional equipment could be retrofitted for autonomous field crop production (Gough, 2018). The HFH model is swarm robotics in the sense that it potentially uses multiple smaller machines to accomplish what a single large machine on conventional farms does. The objective of this study was to identify the potential economic implications of autonomous crop equipment for arable agriculture using a grain-oilseed farm in the United Kingdom as an example. The methodology uses data from the HFH demonstration project and farm-level linear programming (LP) to estimate whole farm profitability of an autonomous cropping system, determine the changes in investment required compared to conventional mechanisation and compare the cost of production. A timely ex-ante economic analysis is needed to: (1) help engineers and entrepreneurs identify the most profitable crop automation alternatives, (2) guide farmers in their decisions about using autonomous crop equipment, and (3) inform policy makers about the costs and benefits of crop robotics.

Farm machines that are mobile and have some autonomy are described with several different terms including: robot, autonomous equipment, automation. Based on the arguments in Kyriakopoulos and Loizou (2006) for this study the word “robot” is reserved for machines with substantial artificial intelligence (AI) decision making capacity, while “autonomous equipment (or machines)” is used for the HFH technology that has autonomy of operation with a predetermined path or itinerary. This study focuses on levels 4 and 5 of the widely used driving automation level scale (SAE, 2018).

Farm LP models have long been used as a means for identifying the portfolio of enterprises and technologies that are the best way of using the farm resources (see e.g. Heady, 1954). This approach has distinct advantages over partial budgeting because (a) it can select a single plan that produces maximum net returns, (b) it allocates the scarce resources (land, labour, machinery) of the farm so as to use them as efficiently as possible in the economic sense and, (c) for complex farming operations, it can quickly and efficiently sort through thousands of alternatives. Numerous books have addressed the subject (e.g. Hazell & Norton, 1986; Kaiser & Messer, 2011), and these models can be adapted for use with farms that include both crop and livestock enterprises (e.g. Morrison et al., 1986). A survey of applications of these types of models can be found in Glen (1987).

Similar farm planning models have been widely used to determine the potential of crop and livestock technology options worldwide. McCarl et al. (1977) describe a model used to help US farmers sort through the genetic, mechanical and chemical technologies that became available in the 1960s and 1970s. Audsley (1981) developed a UK farm LP for evaluation of new machines and farming techniques. Audsley and Sandars (2009) summarized the use of LP and other operations research models in analysis of UK agricultural systems. In recent years farm LP has been used in the UK mainly to identify the most cost-effective environmental management options (e.g. Annetts & Audsley, 2002; MacLeod et al., 2010; Williams et al., 2003).

Farm LP models can also be used to understand the role of risk in farm decision making. Research with mathematical programming models found a limited role for risk aversion in Midwest U.S. agriculture (Brink and McCarl, 1978). Rather than account for risk aversion directly, it has been common practice to handle these through chance constraints for available time when field conditions are suitable (Charnes & Cooper, 1959; Kaiser & Messer, 2011). The HFH-LP uses this good field days approach to modelling risk.

While automation is well established in industrial livestock production, particularly dairy, the use and the economic analysis of autonomous machines for crop production is at its early stages (Lowenberg-DeBoer et al., 2020). Most studies of the economics of crop robotics and autonomous equipment use partial budgeting methods and focus on automation of one crop operation (e.g. weeding, harvesting). Lowenberg-DeBoer et al. (2020) found only three studies that attempted to consider a systems analysis of the economics of crop robotics. The most successful systems analysis is by Shockley and Dillion (2019) who employed an LP model to analyse the economics of using autonomous equipment for maize and soybean production in Kentucky USA. They assumed that all in-house field operations are potentially autonomous, but assumed that contractors would undertake phosphorous and potassium fertilizer application, lime spreading and harvest with conventional equipment operated by human drivers. Parameters for autonomous equipment were based on prototypes developed and tested by their colleagues in the Department of Biosystems and Agricultural Engineering at the University of Kentucky. The analysis compared net returns from using autonomous equipment to the best complement of conventional equipment for a given farm size. Because autonomous equipment for grain production is not yet on the market and the cost of this equipment is unknown, Shockley and Dillion (2019) argued that they cannot determine if autonomous machines would be more cost effective than conventional mechanisation. They reported their key results in terms of the breakeven price of computerised controls that would convert conventional tractors to autonomous. The analysis suggested that relatively small autonomous equipment would have economic advantages for a wide range of farm sizes, but especially for small farms.

This analysis went beyond Shockley and Dillon (2019) as the HFH showed that it is possible to use commercially available global navigation satellite systems (GNSS) and drone autopilot software to retrofit conventional medium scale farm equipment for autonomous operation. The cost and reliability of such equipment is well-defined making it possible to estimate the cost of autonomous field crop equipment. This is particularly relevant as the transition from conventional to autonomous field crop production would initially occur through retrofitted equipment rather than specially designed autonomous equipment. The HFH analysis also goes beyond Shockley and Dillon (2019) to automate all production activities, including fertilizer and lime application, and harvesting.

Given the autonomous crop equipment in process of development and commercialization, and the paucity of systems analysis, the overall objective of this study was to identify the implications of autonomous equipment for the economics of farming, using arable farming in the UK as an example. The specific objectives were to:

1. Estimate the economic feasibility of autonomous equipment for field crop production in the UK,
2. show how autonomous equipment could shift the shape of the UK wheat production cost curve, and
3. identify the implications of this cost curve change for the size and structure of farms in the UK.

The hypothesis is that with autonomous equipment. the UK grain production cost curve would change in two key ways: (1) the cost curve would fall more rapidly for smaller farms and arrive at minimum cost at a smaller farm size than is currently the case, and (2) the UK grain cost curve minimum cost would be closer to (and perhaps below) the import substitution price level. The findings of this study would be applicable to many other arable farming areas of the world where there are physical and economic challenges with small scale farming and the use of large scale conventional equipment.

## The model

The HFH-LP model was based on a well-tested and particularly flexible system for model farming operations known as the Purdue Crop/ Livestock Linear Program (PC/LP) (Dobbins et al., 1994) using the General Algebraic Modelling System (GAMS, ND) modelling language. This model accommodates both crop and livestock production, taking into account the use of crop outputs as feedstuffs. Crop modelling allows for sole crops, multi-year crop rotations and multiple cropping—the raising of more than one crop on the same piece of land within the same year. Categories of resources can be distinguished including owned and hired labour, plots of land with different soil types, and different types of livestock facilities. In many ways, the HFH-LP is similar to the Audsley (1981) UK farm LP, but taking advantage of more recent software.

The HFH-LP model can be expressed in the standard summation notation used by Boehlje and Eidman (1984) as:

1 $$ Max\Pi = \sum\limits_{j = 1}^{n} {c_{j} X_{j} } $$

subject to:

2 $$ \sum\limits_{j = 1}^{n} {a_{ij} X_{j} \le b{\kern 1pt}_{i} } {\text{for}}\,i = 1 \ldots m $$

3 $$ {\text{X}}_{j} \ge {\text{0 for }}j{ = 1} \ldots n $$

where: Π = Gross margin (return over variable cost), *X*_*j*_ = the level of the *j*th production process or activity, *c*_*j*_ = the per unit return (gross margin) to fix resources (*b*_*i*_’s) for the *j*th activity, *a*_*ij*_ = the amount of the *i*th resource required per unit of the *j*th activity, *b*_*i*_ = the amount of the *i*th resource available.

The gross margin of individual activities (*c*_*j*_’s) are total crop sales revenue minus total direct and fixed costs, and can be considered returns to assets. In other words, net returns from the operation equals gross margin minus fixed costs. Government subsidies and taxes are not included in this calculation. In the HFH-LP analysis, the objective function was to maximize gross margin for each set of land, operator labour and equipment. This is a computationally simpler formulation than the integer programming employed by Shockley and Dillon (2019) who include equipment selection within the model. Fixed costs are land, farm facilities, equipment, and compensation for management, risk taking and labour provided by the operator.

As crop yields are dependent on the crop grown during the previous season and the timing of planting and harvest, production activities are modelled as rotations with specific plant and harvest time combinations. For instance, a two crop rotation activity (an *Xj*) might have both crops planted and harvested at their optimal times. Another activity might have both crops planted and harvested later than optimum. Yet another activity might have one crop planted early and the other late. The model uses a simplifying assumption of “steady state” in that it assumes the selected rotations are repeated indefinitely.

Because agricultural activities are often seasonal, the choice of time step is crucial. The HFH-LP assumes a monthly time step. This is a compromise between accurate modelling of the seasonal pattern of work and need to keep the model relatively simple. A quarterly time step would be too coarse. For example, there is an important difference between harvesting oilseed rape (OSR) in July and October, or planting wheat in September or November.

Because of rain and inclement weather, crop activities are constrained to the number of days each month when field work is possible, which is substantially less than the number of calendar days in the month. In each month, the number of good field days can be estimated based on meteorological data. The primary mechanism for modelling risk aversion in the model is the level of probability assumed for the good field days. The standard PC/LP assumption was to use the good field data available in the 17th worst year out of 20 (McCarl et al. 1977). This would be the number of good field day available 85% of the time. The Agro Business Consultants (2018) provide estimates of the number of good field days available in 4 years out of 5 (i.e. 80%). Conventional machine scenarios assume that most field operations occur during daytime (i.e. on average about 10 h per day). The autonomous equipment scenarios assume that all crop operations (seeding, harvesting, fertilisation and spraying) are autonomous and that the autonomous tractors can work 22 h per day with 2 h for repair, maintenance, and refuelling. However, grain harvesting is limited by night dew to 10 h per day.

The primary constraints are:

- *Land*—the sum of land used in production activities is less than or equal to the arable land available. If *q* crops are in a given rotation, the land used for a unit of a rotation is the fractional unit *1/q* of each crop. For example, one hectare of a wheat-oilseed rape rotation is equal to half a hectare of wheat and half a hectare of OSR.
- *Human Labour*—the sum of the labour needed in each month for each crop in the rotation multiplied by the fractional unit (*1/q*) of each crop in a given rotation. The sum of the human labour required must be less than the labour available from the operators, permanent farm labour and temporary farm labour on the number of good field days. Based on HFH experience, human supervision of autonomous equipment is assumed to require 10% of the machine time in the field. This human supervision time parameter is consistent with the 10% supervision time for field work assumed by Engström and Lagnelov (2018) and Lagnelov et al. (2021).
- *Machine Time*—in some cases, the time per day available for certain crop machine operations may be more limited than human operator time. For example, in good weather tillage or plant activities might continue around the clock if humans work in shifts but, because of dew in the UK, combine harvesting of small grains and oilseeds can usually occur only from late morning to dusk. The machine time constraint is that the sum of machine time per crop in a given month on good field days, weighted by the rotation fraction (i.e. *1/q*), must be less than or equal to the amount of machine time available. In the analysis of autonomous equipment for crop production, the machine time is autonomous equipment time required for each crop rotation in each month.
- *Cashflow*—sum of the variable costs for each crop in a rotation in a given month multiplied by the rotation fraction must be less than or equal to the working capital available. In the baseline analysis, this constraint is not limiting.

To focus on the essentials, the initial HFH-LP is specified with a very simple crop rotation and using standard cost estimates from the Nix Pocketbook (Redman, 2018) and The Agricultural Budgeting & Costing Book (Agro Business Consultants, 2018). The primary rotations modelled were winter wheat-oil seed rape (OSR) with a range of timeliness of planting and harvesting. Spring barley-OSR rotations with several timeliness alternatives were included to give the model some flexibility in the timing of field operations. Field operation timing is drawn from Finch et al. (2014) and Outsider’s Guide (1999). Equipment timeliness estimates and other machine relationships are from Witney (1988). All crops are assumed to be direct drilled. Key baseline assumptions are shown in Table 1 and further described in detail in the Electronic Supplementary Materials.

**Table 1:** Key baseline assumptions for HFH and conventional farm model

Parameter	Unit	Value
Soil type		Very slightly stony sandy loam (Salop series)^a^
Crop yields		
Wheat	t/ha	9.1
Oilseed rape (OSR)	t/ha	3.75
Spring barley	t/ha	6.0
Crop prices		
Wheat	£/t	155
OSR	£/t	509
Spring barley	£/t	163
Crop direct costs		
Wheat	£/ha	537
OSR	£/ha	509
Spring barley	£/ha	334
Machinery investment costs		
28 kW HFH autonomous set	£	91 162
28 kW conventional set	£	67 900
112 kW conventional set	£	359 500
221 kW conventional set	£	723 500

^a^Soil Survey of England and Wales (1984)

To explore the implications of the baseline models solutions were generated for each combination of four farm sizes and the four equipment sets available. The farm sizes (assuming all are 90% arable) included:

- A 66 ha farm—this is the average farm size in the West Midlands of the UK (DEFRA 2018a).
- A 159 ha farm—this is the average size of cereals farms in England (DEFRA 2018b).
- A 284 ha farm—this is the average size of cereals farms over 100 ha in England (DEFRA 2018b).
- A 500 ha farm—this is an arbitrary larger farm size.

The equipment sets included:

- HFH sized equipment (28 kW tractor) with human drivers.
- HFH autonomous equipment (28 kW tractor).
- Smaller conventional equipment (112 kW tractor).
- Large conventional equipment (221 kW tractor).

## Baseline results

Summaries of the initial solutions are presented in Table 2. The solutions listed in Table 2 leave no unplanted area because in normal circumstances farmers will prefer a plan that uses their entire resource base. The solutions assume one full time operator, temporary labour available on an hourly basis, and that conventional equipment is typically operated at up to 10 h per day. The superscript in the scenario name indicates the number of equipment sets that are needed to farm the specified area. For example, “Autonomous^3^” means that it requires three sets of the HFH equipment to farm the 450 arable ha under the assumptions used.

**Table 2:** Summary of initial HFH-LP solutions for representative farm sizes with temporary labour available

Scenario	Arable area (ha)	Labour hired (days)	Operator time (days)	Whole farm gross margin (£/year)	Return to operator labour, management and risk taking (£/year)	Wheat cost of production with operator labour cost allocated (£/t)
Conv 28 kW	59.4	0	79	47 048	15 846	168
Conv. 28 kW^2^	143.1	72	118	107 759	36 344	150
Conv. 28 kW^3^	255.6	195	144	187 237	64 923	140
Conv. 28 kW^4^	450.0	411	186	302 920	99 321	137
Autonomous	59.4	0	26	47 048	12 301	140
Autonomous	143.1	8	54	112 691	46 891	125
Autonomous^2^	255.6	50	62	198 587	78 ,340	122
Autonomous^3^	450.0	121	76	347 015	141 936	118
Conv.112 kW	59.4	0	28	47 048	−26 001	212
Conv.112 kW	143.1	0	68	112 243	8142	157
Conv.112 kW	255.6	31	89	200 017	54 178	136
Conv.112 kW	450.0	108	104	331 989	63 017	140
Conv.221 kW	59.4	0	16	47 048	−70 973	288
Conv.221 kW	143.1	0	39	113 343	−35 731	182
Conv.221 kW	255.6	1	69	202 371	11 560	152
Conv.221 kW	450.0	35	87	353 677	90 743	131

The superscript under scenario indicates the number of autonomous equipment sets

Table 2 shows that under the assumptions used, the small conventional equipment is quite profitable, but requires substantial amounts of hired labour. While tractor drivers are easier to hire in the UK than workers for hand weeding, vegetable harvesting or other farm manual labour, it is not obvious that the amount of labour needed could be hired at the average wage of £9.75/h assumed in this analysis. Because grain production is already highly mechanised it may be converted to autonomous production more easily than horticulture where many production processes are still manual.

In terms of operator time (Table 2), the results indicate that the use of small scale conventional equipment also requires the operator to spend a substantial amount of time driving a tractor or combine. For example, if full time work is about 220 days per year, then the 450 ha arable farm Conv. 28 kW^4^ scenario would require the operator to spend 85% of their time operating equipment, leaving very little time for management, marketing and other farm tasks.

With the assumption that supervision of the autonomous equipment requires about 10% of the equipment field time, the total operator time commitment to crop operations with autonomous equipment is roughly equivalent to that of the scenarios with large scale human- operated conventional equipment. This occurs even with a modest 10% supervision time because the small autonomous equipment requires up to five times longer to cover the same area than the larger conventional equipment and the assumption is that the 10% applies to each autonomous unit. Experience will show whether the 10% supervision time based on HFH experience will be typical of other autonomous equipment farms.

For the autonomous equipment farming scenario, the bulk of the human time is devoted to hauling grain from the field to the farmstead or market during harvest in July, August and September. For example, in the 284 ha autonomous equipment scenario, 45% of the annual operator time and all of the hired labour is devoted to grain hauling from the field to the farmstead or market. This hired labour represents a cash cost of £7724, but even more important than the expense is the difficulty of filling this harvest time spike in labour demand. This suggests that one technical priority for autonomous equipment agriculture should be to develop a system in which either the grain transport from field to farmstead/market is automated (i.e. self-driving lorries), or where grain is stored in the field until it is used or goes to market.

Because the direct costs and yields are assumed to be the same across all scenarios, the gross margins are similar at each farm size (Table 2). For the smallest farm, gross margins are identical for each equipment scenario (i.e. £47 048) because all four equipment scenarios are able to plant and harvest the wheat/OSR rotation in the optimal periods. For the larger farms, the gross margin differences occur because: (1) some planting and harvesting occurs in non-optimal months, (2) equipment and labour constraints force less profitable spring barley into the crop mix (see the autonomous scenario for the 255.6 ha arable farm), and (3) some solutions use more temporary labour. Notably, the large conventional equipment produces the highest gross margins for all farm sizes, but returns to operator labour, management and risk taking are lower than the autonomous farm after taking into account the costs of machinery ownership.

While most of the discussion of the economics of autonomous crop equipment has been focused on reducing the human labour requirements and cost, this analysis suggests that there may be an equally important impact on reducing equipment investment costs by using smaller equipment more intensively. The equipment investment for the large conventional farm is estimated at £723 500 and for the conventional farm with the 112 kW tractor £389 500. This assumes the purchase of new equipment. The estimated new equipment investment for one set of the autonomous equipment is £91 162, with £23 262 of that being the real-time kinematic GNSS and modified drone software. For the 450 ha farm, the equipment investment for the autonomous equipment farm is £273 486 (three sets of the HFH equipment) or only 39% of the estimated investment for the 221 kW tractor conventional farm, which provides the minimum wheat production cost among conventional alternatives. By using smaller equipment more intensively, the autonomous equipment farm is able to substantially reduce capital costs.

In this analysis, the return to operator labour, management and risk taking is highest for the autonomous equipment, except for the small scale conventional equipment on the smallest farm. This occurs because the operator is assumed to be full time on the farm (i.e. operator compensation is not deducted from the return estimate) and because of the added investment to retrofit the equipment for autonomous operation. For the larger farms, the autonomous scenario has the highest return to the operator.

Cost curves are used in this study to analyse farm size and structure issues, because much of the debate in economics about farm economies of size is in terms of cost of production (Miller et al., 1981). The cost of wheat production is estimated because wheat is widely grown and costs are well documented in many countries. Economic theory indicates that farms which operate at the farm size with the lowest unit cost of production will be more successful and over time the structure of the farming industry will tend toward that lowest unit cost of production farm size (Duffy, 2009; Hallam, 1991; Miller et al., 1981). Economic research in the 1960s and 1970s in North America suggested that for many farm products the long run average cost curve is “L” shaped, where unit costs are higher on small farms. These costs fall as farm size grows until the long run average cost curve levels out at minimum cost. It has also been hypothesized that the cost curve would eventually rise for very large farming operations because of management complexity, but in practice that has not been widely observed with conventional crop technology. This research argues that a broader range of economically viable farm sizes may be observed when the bottom of the cost curve is nearly flat. The key empirical issue then is at what farm size is minimum cost achieved? The hypothesis is that autonomous equipment would allow a farmer to achieve minimum costs of production at a smaller scale than conventional equipment would. In terms of the cost curve, this means that the autonomous equipment cost curve would arrive at a relatively flat bottom at a much smaller scale than the conventional cost curve.

The wheat production cost estimates include all direct costs and indirect costs for machinery, farm infrastructure and operator compensation prorated to the time devoted to field activities, plus 20%. The extra 20% is assumed to be needed for management and marketing. The operator compensation estimate is from the 2016 Farm Manager Survey (Redman, 2018, p. 166). That estimate is £52 238 in monetary compensation, plus £12 530 in non-cash benefits including rent free accommodation, mobile phone and use of a motor vehicle, totalling to £64 768 per annum.

**Fig. 1:**
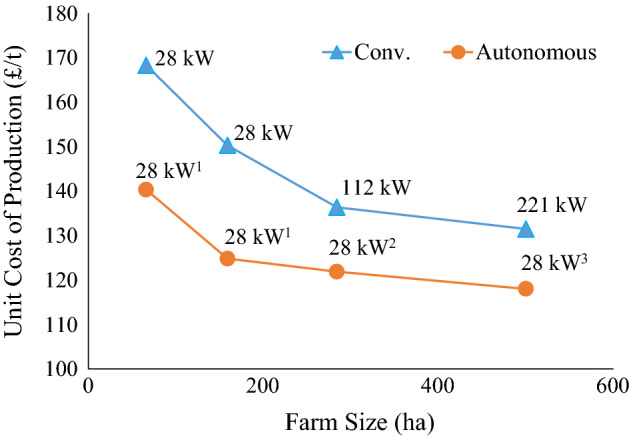
Wheat unit production cost (£/t) for farms equipped with conventional (triangles) or autonomous equipment (circles) for a range of farm sizes and with operator labour cost allocated. Superscripts indicate the number of autonomous equipment sets

A chart of the wheat production costs estimated using HFH-LP takes an approximate “L” shape (Fig. 1) with the cost curve for autonomous equipment below the conventional cost curve. The cost curves assume that for conventional equipment, farmers will choose the equipment size that minimises the cost, such that the conventional curve is at the minimum cost over the three equipment scenarios. The data labels at each point are the tractor size for the least cost equipment set for that farm size. The number of autonomous units used for the least cost equipment sets is shown via a superscript. The conventional and autonomous equipment cost curves have similar shapes, but that may be because costs are estimated for a very limited number of equipment scenarios. If there were more equipment scenarios, the estimate would be more likely to indicate differences in the cost curve shape. Assumptions about allocation of farm operator time and costs may also affect the shape.

International comparisons of agricultural costs of production are fraught with difficulties because of exchange rates, explicit and implicit government subsidies, differing production practices, quality differences and other factors, but the *agri benchmark cash crop network* (http://www.agribenchmark.org/home.html) has monitored comparable costs for major production countries. Balieiro (2016) presented wheat production costs for 2008–2015 across eight countries which indicated a range from £123-£192/t (GBP = US$1.30), with UK costs of production at the upper end of that range. Estimates for Russia and Ukraine are as low as £62-£77/t. Most of the recent UK wheat imports were from Canada, Germany and France with the costs of production estimated between about £123 and £154/t. With wheat cost of production on the autonomous equipment farm under £120/t, UK wheat would be much more competitive with imported wheat than that produced on the conventional farm. Analysis is needed to determine if other UK farm products would be more internationally competitive with use of autonomous equipment.

The advantage of using autonomous equipment would be higher for crops and production systems that require more field operations and more labour. For example, one of the main economic constraints on organic production has been the high cost of manual weeding; autonomous mechanical weeding could make organic production substantially more cost competitive. Similarly, autonomous equipment may help reduce the cost disadvantage of non-standardized heirloom varieties with desirable nutritional or consumer attributes, but which could not easily be harvested mechanically.

An additional benefit of using smaller equipment sets, whether they be conventional or autonomous, would be their ability to better handle in-field obstacles (e.g. trees, power poles) and smaller irregularly sized fields. With agricultural intensification and the continually increasing size of equipment sets in the UK, there has been clear evidence of ongoing field enlargement (hedge removal) and tree clearing (Robinson & Sutherland, 2002). With a much-reduced impact of smaller and irregularly sized fields on the operating efficiency of smaller equipment sets, and as this study indicates, comparable costs of production and more profitable scenario outcomes, adoption of such systems would reduce or even lead to a reverse in the impacts of agricultural intensification and large scale mechanisation.

The HFH-LP also provides information on the marginal values or “shadow prices” of the various farm resources. These shadow prices indicate the net effect on the objective function (i.e. maximum profit or gross margin achieved) from a small change in a constraint limit. For example, the HFH-LP for the autonomous scenario for the 284 ha farm shows that tractor time is limiting in October and November during drilling of winter crops. The maximum number of 8 h tractor work days available in October is 52.25 (= 19 good field days × 2.75 workdays per field day if working 22 h per day). The maximum number of 8 h tractor work days available in November is 41.25 (= 15 good field days × 2.75 workdays per field day if working 22 h per day). The shadow price of tractor time is £623.72/work day; that means the total gross margin could be increased by £625.72 if one more 8 h day of autonomous tractor time could be found. The shadow price of November tractor time is lower; it is only £41.81/workday reflecting the lower average yields and profits if wheat is planted in November rather than October.

Similarly, combine time is limiting in July and August for the Autonomous scenario for the 284 ha farm. The shadow price of combine time in July is £1486.64/workday and in August £1377.96/workday. As with the tractor, the units are 8 h work days. Defining the shadow prices for different constraints can help technology developers target the highest value innovations.

## Limitations

The HFH LP is a preliminary model of how autonomous equipment would affect field crop decisions in the UK. The analysis depends on several non-technical assumptions:

1. The ownership model of acquiring farm equipment services is relevant for autonomous machines. Alternative service provider, rental and leasing approaches are widely discussed by robotics researchers and entrepreneurs.
2. Continuous on-site human supervision is not required for the autonomous equipment farm. Currently, on-site human supervision of autonomous agricultural machines is generally required in EU countries under the EC Machine Directive 2006/42/EC (EC, 2010) and is required in the UK for drones. On-site human supervision of autonomous equipment is also required in the US State of California [California Code of Regulations, Title 8, Sect. 3441(b)]. A 100% on-site supervision requirement removes much of the cost savings when farming with autonomous equipment, but sensitivity testing indicates that the overall results of this analysis hold for up to 50% on -site human supervision.
3. Public liability and standard commercial insurance is available for the farm with autonomous equipment at comparable cost to conventional farms.
4. That the commercial manufacturing and sale of autonomous equipment achieves economies of scale (i.e. autonomous equipment is widely purchasable and serviced).

As experience with autonomous crop equipment accumulates and assumptions are clarified, the results of this baseline study may be substantially modified. For example, on-site human supervision may make autonomous equipment uneconomic on smaller farms because it would mean covering the costs of both the human supervisor and the autonomy hardware and software. If continuous on-site human supervision is required, in many cases it may be less costly to have the human drive the equipment. If insurance for autonomous equipment turns out to be high cost, that could stop its use on farms. Depending on the business model used, service provider, rental and leasing options could increase or reduce timeliness, effectiveness and costs for autonomous equipment for certain field operations, and either enhance or reduce their profitability.

## Conclusions

This study provides an economic analysis that supports the hypothesis that autonomous crop equipment has the potential to dramatically alter the economic environment for arable farms. The estimated wheat production cost curve with autonomous equipment achieves almost minimum levels at a smaller farm size than the conventional equipment cost curve. The estimated wheat production cost with autonomous equipment is largely within the range of most exporting countries and for medium scale farms below that cost range. The ability to achieve near minimum production costs at relatively smaller farm sizes, and with a modest equipment investment, means that the pressure for farming businesses to continually seek economies of scale (i.e. to “get big or get out”) is diminished. This provides the opportunity for modest size grain enterprises to become profitable instead of being a lifestyle choice. With reducing the need for labour and equipment investment, those modest sized grain enterprises could be combined with livestock, on-farm value added activities or off farm employment to provide enough income for family needs. Costs of production that are internationally competitive mean that there is a reduced reliance on government subsidies for survival and greater independence for farmers. The ability of autonomous equipment to achieve minimum production costs, even on small, irregularly shaped fields, can reduce the environmental impacts of grain production. It has the potential to reduce the pressure to remove hedges, fell infield trees and enlarge fields, as well as maintain better soil structure and fertility.

## Data and materials availability

All data is available in the main text or the supplementary materials.

## Coding availability

General Algebraic Modeling System (GAMS) code in Appendix A of the supplementary materials.

## Supplementary Information

Below is the link to the electronic supplementary material.

Supplementary file1 (DOCX 19 kb)

Supplementary file2 (XLSX 36 kb)

Supplementary file3 (XLSX 36 kb)

Supplementary file4 (XLSX 36 kb)

Supplementary file5 (XLSX 36 kb)

Supplementary file6 (DOCX 52 kb)
