# Supplementary material for: Economics of autonomous equipment for arable farms
Source: Precis Agric. 2021 May 27;22(6):1992–2006. doi: 10.1007/s11119-021-09822-x (PMC8154546; doi:10.1007/s11119-021-09822-x)
Supplement: Supplementary file 1 — Supplementary file1 (DOCX 19 kb) [file 11119_2021_9822_MOESM1_ESM.docx]

Appendix A – UK-Wheat-OSR-Barley_EditedRobot3-20190117.gms – The GAMS source code for the preliminary version of the HFH-LP

* The origins of this model are from Preckel et al. (2019), and were modified by

* Lowenberg-DeBoer et al. (2019). This program may be distributed or further modified

* provided that this comment remains and is updated according to the future modifications

* and provided that any modified program continues to be freely distributed.

* Citations:

* P. Preckel, C. Fontanilla, J. Lowenberg-DeBoer, J. Sanders, “Orinoquia agricultural linear

* programming model – documentation.” (Colombia Purdue Partnership, Purdue University,

* <https://www.purdue.edu/colombia/partnerships/orinoquia/docs/OrinoquiaLPDoc.pdf>,

* 2019).

* Lowenberg-DeBoer, James, Karl Behrendt, Richard Godwin and Kit Franklin, “The Impact of

* Swarm Robotics on Arable Farm Size and Structure in the UK.” – paper presented at the

* Agricultural Economics Society (AES) Conference, April 2019, Warwick, UK.

* Note that if you change the name of the data spreadsheet, then the name

* of the first Excel file in the line below to the name of your new file.

$call copy UK-Wheat-OSR-Barley_Conv38hp_20190125.xlsx Orinoquia_Tables.xlsx

* If you would like to see the equations, increase limrow in the line below.

* If you would like to see the activities, increase limcol in the line below.

Option limrow=0,limcol=0 ;

* Note all of the data are read from the spreadsheet; so not data appears in

* this program.

Sets

t(*) Time periods

e(*) Enterprises

c(*) Commodities

l(*) Land type ;

Scalars

flab Family labor available (no. of workers)

plab Permanent labor available already employed (no. of workers)

trac Autonomous Tractors available (man days)

comb Autonomous Combines available (man days)

thlab Maximum hired temporary labor in each period (man days)

phlab Maximum additional permanent labor hired (man years)

twlab Temporary wage (GBP per 8 hour man day)

pwlab Permanent worker wage (GBP per man year)

initcash Initial cash available (GBP)

intrst Monthly interest rate

mxborrow Borrowing constraint in GBP ;

* The following does imports of data from Gams_Tables.xlsx.

$onecho > tasks.txt

set=t rng=Scalars_and_Parameters_(R)!f4 dim=1 rdim=1

set=e rng=Labor_use_(R)!b2 dim=1 rdim=1 maxdupeerrors=50

set=c rng=Commodity_produced_(R)!c2 dim=1 rdim=1 maxdupeerrors=50

set=l rng=Commodity_produced_(R)!a2 dim=1 rdim=1 maxdupeerrors=50

par=flab rng=Scalars_and_Parameters_(R)!c3 rdim=0 cdim=0

par=plab rng=Scalars_and_Parameters_(R)!c4 rdim=0 cdim=0

par=trac rng=Scalars_and_Parameters_(R)!c5 rdim=0 cdim=0

par=comb rng=Scalars_and_Parameters_(R)!c6 rdim=0 cdim=0

par=thlab rng=Scalars_and_Parameters_(R)!c7 rdim=0 cdim=0

par=phlab rng=Scalars_and_Parameters_(R)!c8 rdim=0 cdim=0

par=initcash rng=Scalars_and_Parameters_(R)!c9 rdim=0 cdim=0

par=intrst rng=Scalars_and_Parameters_(R)!c10 rdim=0 cdim=0

par=mxborrow rng=Scalars_and_Parameters_(R)!c11 rdim=0 cdim=0

par=twlab rng=Scalars_and_Parameters_(R)!c12 rdim=0 cdim=0

par=pwlab rng=Scalars_and_Parameters_(R)!c13 rdim=0 cdim=0

$offecho

$call GDXXRW Orinoquia_Tables.xlsx trace=3 @tasks.txt

$GDXIN Orinoquia_Tables.gdx

$LOADDC t,e,c,l,flab,plab,trac,comb,thlab,phlab,initcash,intrst,mxborrow,twlab,pwlab

$GDXIN

Set

fnt(t) Final time period ;

fnt(t) = Yes$(ord(t) eq card(t)) ;

* Display those sets and scalar values in case we need to verify the data got

* read in all right.

Display t,fnt,e,c,l,flab,plab,trac,comb,thlab,phlab,initcash,intrst,mxborrow,twlab,pwlab;

* Declare the parameters whose values will also be read from the spreadsheet.

Parameters

lnd(l) Land of type l available (ha)

gfd(t) Good field days available in period t (days per period)

wu(l,e,t) Labor use of enterprises by period

tu(l,e,t) Autonomous tractor use of enterprises by period

comu(l,e,t) Autonomous combine use of enterprises by period

cu(l,e,t) Cash use for enterprises by period

lu(l,e,t) Land use of enterprises by period (ha per period)

entcom(l,e,c,t) Quantity commodity c produced per unit of enterprise e on land l

* fu(l,e,c,t) Commodity use of enterprises by period (intermediate inputs)

sprc(c) Selling prices for commodities (GBP per unit)

lobd(l,e) Lower bounds on commodities by enterprise (ha)

upbd(l,e) Upper bounds on commodities by enterprise (ha) ;

* Import parameter values.

$onecho > tasks.txt

par=lnd rng=Scalars_and_Parameters_(R)!b16 dim=1 rdim=1

par=gfd rng=Scalars_and_Parameters_(R)!f4 dim=1 rdim=1

par=sprc rng=Scalars_and_Parameters_(R)!j4 dim=1 rdim=1

par=wu rng=Labor_use_(R)!a1 rdim=2 cdim=1

par=tu rng=Trac_use_(R)!a1 rdim=2 cdim=1

par=comu rng=Comb_use_(R)!a1 rdim=2 cdim=1

par=cu rng=Cash_use_(R)!a1 rdim=2 cdim=1

par=entcom rng=Commodity_produced_(R)!a1 rdim=3 cdim=1

par=lu rng=Land_use_(R)!a1 rdim=2 cdim=1

* par=fu rng=Commodity_use_(R)!a1 rdim=3 cdim=1

par=lobd rng=Scalars_and_Parameters_(R)!n4 dim=2 rdim=2

par=upbd rng=Scalars_and_Parameters_(R)!s4 dim=2 rdim=2

$offecho

$call GDXXRW Orinoquia_Tables.xlsx o=Orinoquia_Tables_par.gdx trace=3 @tasks.txt

$GDXIN Orinoquia_Tables_par.gdx

$LOADDC lnd,gfd,sprc,wu,tu,comu,cu,entcom,lu,lobd,upbd

$GDXIN

* Display those parameter values in case we need to verify the data got

* read in all right.

Display lnd,gfd,sprc,wu,tu,comu,cu,entcom,lu,lobd,upbd ;

* Set the enterprise-land type mapping based on whether the enterprise

* produces any commodity in any time period on the specific land type.

* If it doesn't produce anything, it gets supressed.

Set

el(e,l) Enterprise-land type included if enterprise has output on land l ;

el(e,l) = Yes$(sum((c,t),entcom(l,e,c,t)) gt 0) ;

el(e,l)$(lnd(l) eq 0) = No ;

Display el ;

Positive Variables

produce(l,e) Produce enterprise e on land type l (ha)

sell(c,t) Sell commodity c in period t (commodity units)

phire Permanent labor hired (man years)

thire(t) Temporary labor hired in period t (man days)

save(t) Cash stored from period t to t+1 (GBP)

borrow(t) Cash borrowed in period t and repaid in period t+1 (GBP) ;

Variables

netret Net return to the farm (GBP) ;

Equations

land(l,t) Limit on land use for land of type l in period t (ha)

labor(t) Define amount of labor to hire in period t (man days)

autotrac(t) Define autonomous tractor use in period t (man days)

autocomb(t) Define autonomous combine use in period t (man days)

comuse(c,t) Sources and uses for commodity c in period t (commodity units)

cash(t) Sources and uses of cash in period t (GBP)

nrobj Net return objective ;

land(l,t) ..

sum(e$el(e,l),lu(l,e,t)*produce(l,e)) =l= lnd(l) ;

labor(t) ..

sum(el(e,l),wu(l,e,t)*produce(l,e)) =l=

(flab+phire)*gfd(t) + thire(t) ;

autotrac(t) ..

sum(el(e,l),tu(l,e,t)*produce(l,e)) =l=

(trac)*gfd(t) ;

autocomb(t) ..

sum(el(e,l),comu(l,e,t)*produce(l,e)) =l=

(comb)*gfd(t) ;

comuse(c,t) ..

sell(c,t)

=l=

sum(el(e,l),entcom(l,e,c,t)*produce(l,e)) ;

cash(t) ..

sum(el(e,l),cu(l,e,t)*produce(l,e))

+ phire*pwlab/card(t)

+ thire(t)*twlab + save(t)

+ borrow(t-1)*(1+intrst)

+ initcash$fnt(t) =l=

initcash$(ord(t) eq 1) + sum(c,sprc(c)*sell(c,t))

+ save(t-1) + borrow(t)$(not fnt(t)) ;

nrobj ..

netret =e= sum(fnt,save(fnt)) ;

* Set bounds on individual variables.

produce.lo(l,e) = lobd(l,e) ;

produce.up(l,e) = upbd(l,e) ;

thire.up(t) = thlab ;

phire.lo = plab ;

phire.up = plab + phlab ;

borrow.up(t)= mxborrow ;

Model finca / land,labor,autotrac,autocomb,comuse,cash,nrobj / ;

option lp=cplex ;

finca.optfile=1 ;

Solve finca using lp maximizing netret ;
