# Supplementary material for: Economics of autonomous equipment for arable farms
Source: Precis Agric. 2021 May 27;22(6):1992–2006. doi: 10.1007/s11119-021-09822-x (PMC8154546; doi:10.1007/s11119-021-09822-x)
Supplement: Supplementary file 6 — Supplementary file6 (DOCX 52 kb) [file 11119_2021_9822_MOESM6_ESM.docx]

Supplementary Materials for:

**Economics of Autonomous Equipment for Arable Farms**

James Lowenberg-DeBoer*, Kit Franklin, Karl Behrendt, Richard Godwin

Harper Adams University, Newport, Shropshire, UK TF10 8NB.

*Correspondence to: jlowenberg-deboer@harper-adams.ac.uk

**This file includes:**

Supplementary Text

Tables S1 to S5

**Other Supplementary Materials for this manuscript include the following:**

Appendix A – General Algebraic Modeling System (GAMS) code

Appendices B-E – Input parameters for scenarios

Supplementary Text – Hands Free Hectare (HFH) Linear Programming (LP) Model Assumptions and Parameter Estimation

The HFH-LP model was based the Purdue Crop/ Livestock Linear Program (PC/LP) (Preckel *et al.* 1992; Dobbins *et al.* 1990; Dobbins *et al.* 1992; Dobbins *et al.* 1994). This system was used from the mid-1990s through to 2010 as an analytical tool for Purdue’s Top Crop Farmer Workshop.  Farmers from across the Midwestern United States came to Purdue each summer and developed linear programming models for their farms to evaluate alternative technologies and resource investments. An updated version of the PC/LP system has been developed in the General Algebraic Modelling System (GAMS, ND) modelling language. This GAMS version was used by the Purdue University Orinoquia Initiative to help the government of Colombia evaluate proposals for agricultural development in the Orinoco River basin. Orinoquia LP model is described at by Preckel *et al.* (2019) and Fontanilla (2019). The HFH-LP model is a modified version of the PC/LP model using the GAMS software.

Key baseline assumptions in estimating HFH LP parameters are:

- ***Rotations -*** The farm is in a wheat-oilseed rape (OSR) rotation, with a spring barley-OSR rotation available to provide some timing flexibility alternatives. It is assumed that the optimum plant and harvest dates are (Finch et al. 2014, Outsider’s Guide 1999):
  - ***Wheat –*** plant in October, harvest in August
  - ***OSR –*** plant in September, harvest in July
  - ***Spring Barley –*** plant in March, harvest in August

Because of weather, equipment limitations and other factors, farmers cannot always do field operations at the optimum time. In spite of the yield penalty, they sometimes expect to plant and/or harvest before or after the optimum. The plant and harvest alternatives included in the model are listed in Table S1.

The activities names can be deciphered as follows - The first item and immediately after the slash (“/”) is the crop in the two year rotation. The month after each crop is when it is planted. The activity names that end in “Late” have harvest a month later than optimum for both crops. The yield loss from seeding wheat or barley at other than the optimal time is estimated based on Witney (1988) at: wheat seeding in November, 4.18% yield loss; barley planted in February, 7.66% loss; barley planted in January, 31.71% loss. Because Witney does not provide estimates for untimely seeding of OSR, the wheat estimate is used. Witney discusses losses from untimely harvesting, but does not provide a numerical estimate. This preliminary analysis assumes a 10% loss from one month late harvest for all crops.

- ***Direct drill*** - All crops are assumed to be planted with direct drill methods. This is the simplest from the equipment perspective and was the seeding method used by HFH. In the future, activities with tillage alternatives can be developed.
- ***Prices, Yields, Direct Costs*** - Expected prices, yields and direct costs expected for the 2020 harvest were taken from the Agricultural Budgeting & Costing Book (2018).
  - Winter wheat for feed – 9.1 tons/ha - £155/ton - £537/ha direct cost
  - OSR – 3.75 tons/ha - £325/ton - £509/ha direct cost
  - Spring barley for feed – 6 tons/ha - £163/ton - £334/ha direct cost

The direct costs are seed, fertilizer, plant protection and miscellaneous inputs.

- ***Transport on Public Roads*** - A human driver is needed to transport the grain from the field to the farmstead or market on public roads. In the United Kingdom (UK) only the smallest farms would have contiguous fields allowing transport of grain to the farmstead without recourse to the public roads. This will probably be the case until driverless vehicles are perfected and allowed on UK roads. A sensitivity test should be done to estimate the impact of this need for a human driver for grain hauling.
- ***Hired Labor*** - Short term (hourly labor) is available at £9.75/hour and based on UK labor law is limited to 48 hours per week (Agro Business Consultants, 2018) Human, machine and autonomous equipment time in the model is measured in eight hour increments. The eight hour day was take over from the Orinoquia analysis. A sensitivity test should be done for the cases in which labour is not available.
- ***Equipment*** - Equipment was priced either based on what HFH paid or from Agro Business Consultants (2018). The model operates for a fixed equipment set. Four sets will be included in the initial analysis, including:
  - HFH equipment with a human operator. This includes a 38 hp tractor, 1.5 m drill and combine with a 2 m head.
  - HFH autonomous equipment.
  - The smallest direct drill equipment set that can be estimated from Agro Business Consultants (2018) with a 150 hp tractor, 3 m drill and combine with 4.5 m head. The tractor size was estimated assuming about 50 hp per meter of direct drill.
  - A larger direct drill equipment set with a 296 hp tractor, 6 meter drill and combine with 7.5 m head.

Estimated cost of each equipment set is in Table S2. The tractor horsepower requirement is estimated at approximately 50 hp/meter of direct drill. HFH used a donated plot combine. A farmer would need a commercial combine, but farm equipment dealers in the UK no longer handle combines of this size. Consequently, at the combine cost was based on the price of a combine built by a European manufacturer for the Asian market at £28,000. The HFH estimates in Table S2 are for conventional small equipment retrofitted to be autonomous. The main items needed for the retrofit are listed in Table S3. Because it was not “autosteer ready”, the HFH tractor used all the items on the list for a total of £9,345. An autosteer ready tractor would not need the items listed under control adaptions (total of £2,058). The combine required all the tractor items, plus the laser and actuators listed at the bottom for a total of £13,917. Driver’s seats and steering wheels are retained on the autonomous equipment to allow a human driver to move the equipment on public roads. The scenario with the HFH sized equipment with a human driver has the same costs, except for the Table S3 autonomous retrofit hardware and software.

- ***Field work rates*** - Rather than take field work rates from Agro Business Consultants (2018), the field time required for crop activities is calculated in Table S4 with assumptions about overlap, field speed and field efficiency. This provides a comparable estimate for conventional and autonomous equipment. In the baseline analysis all equipment is assumed to overlap 10%. This is a common baseline assumption in the analysis of global navigation satellite systems (GNSS) autosteer technology (Lowenberg-DeBoer 1999; Griffin et al. 2005; Ortiz et al. 2013)*.* Future analysis might reduce that overlap for the autonomous equipment and conventional equipment with GNSS guidance. The HFH field speeds are based on HFH experience. It is assumed that field efficiency is 70% based on the discussion in Witney (1988). Field time required each month by crop is listed in Table S5.
- ***Good Field Days*** – For the preliminary analysis good field days per month are from Agro Business Consultants (2018), January, 16; February, 14; March, 19; April, 20; May, 22; June, 23; July, 22; August, 22; September, 19; October, 19; November, 15; and December, 16. These represent the number of days available at an 80% probability that are available four years out of five. It is assumed that drilling, fertilizer spreading and spraying can occur around the clock. Harvesting is assumed possible from midmorning to dusk or about 10 hours per day in late summer. Analysis of current meteorological data would reveal if these good field days are still relevant and how they might differ by region. Time commitments by crop and operation are in Table S4.
- ***Land*** - The model is run for the following farm areas:
  - 66 ha – Average farm size in the West Midlands of the UK (DEFRA 2018a).
  - 159 ha – Average size of cereals farms in England (DEFRA 2018b).
  - 284 ha – Average size of larger cereals farms in England (DEFRA 2018b). Average of cereals farms over 100 ha.
  - 500 ha – An arbitrary larger size farm

For this analysis the farm is assumed to be in the West Midlands and the farm area is assumed to be 90% tillable with the remainder used for lanes, hedgerows, drainage ditches, farmstead, etc. Each farm size is run for each equipment set. The autonomous scenarios with larger farm hectarage are run with multiple sets of the small equipment approximating a swarm scenario. It is assumed that in the longer run, the farmer would choose the equipment set that is the right size for the farm. In the initial analysis no contractor services were assumed. Obviously, equipment sizes are lumpy; they come in fixed sizes. In the longer run multiple equipment sets could be created to smooth the cost estimates.

- ***Return to Operator Labour, Management and Risk Taking*** – The HFH-LP maximizes return over variable cost. To calculate the return to operator labour, management and risk taking, the cost of land, equipment and faculties must be deducted. Following the principles in Witney (1988), the equipment costs were estimated as follows:
  - ***Depreciation*** – Straight line depreciation assuming a 10 year life for the drill, sprayer and fertiliser spreader, and 20 years for the tractor, combine and other equipment.
  - ***Opportunity cost of capital*** – 3% of the original investment. In normal times 3% would be very low, but Agro Business Consultants (2018) indicates that UK commercial lending rates are around that level.
  - ***Insurance*** – Set as a percentage of the original investment. For the preliminary analysis that percentage was 1% for the tractor and combine, and 0.25% for the other equipment.
  - ***Repair and Maintenance*** – Set at 2% of the original investment
  - ***Fuel and lubricant*** – Set at 2% of the original investment for the tractor and combine.

Following Agro Business Consultants (2018) example for cereal farms, the following fixed costs were assumed for the preliminary analysis:

- - ***Land Rent*** – The average land rent is £216/ha.
  - ***Property and Building Repairs*** - £3/ha
  - ***Professional Fees and Subscriptions*** - £25/ha
  - ***Water, Electricity, etc***. - £55/ha
  - ***Building Depreciation*** - £22/ha
  - ***Miscellaneous Fixed Costs*** - £50/ha
- ***Input Resupply*** – The analysis assumed that refilling equipment with fuel, seed, fertilizer and pesticides is part of the normal work load for the conventional and part of the human supervisory time for the autonomous system.
- ***Finance*** - Cashflow, working capital and financing are not binding constraints. The GAMS code inherited from the Orinoquia Initiative includes working capital constraints, so this can be explored in subsequent iterations of the analysis.

***GAMS –*** The General Algebraic Modeling System (GAMS) was developed by the World Bank. It is the most commonly used mathematical programming software for agricultural research. A demo version of the GAMS software is available at:

<https://www.gams.com/download/>

The demo version will solve problems with up to 300 constraints and is adequate for learning. The preliminary version of the HFH-LP is designed to run on the demo version. The full version of GAMS is available at a significant charge. There are some GAMS learning materials on the GAMS site:

<https://www.gams.com/products/introduction/>

Following the example of the Orinoquia LP model, the HFH-LP software has two parts. The first of these is the GAMS source program. The source program should be modified with care, except for the third line of the program as will be explained below. The second part of the inputs is in the form of a spreadsheet that contains a series of worksheets that contain the data for the LP.

As mentioned, the GAMS source file should be modified with care, except for one exception – the third line of the program. This line is used to link the GAMS program with the correct data spreadsheet. For the example when the GAMS source file is named UK-Wheat-OSR-Barley_EditedRobot3-20190117.gms and the data spreadsheet is named UK-Wheat-OSR-Barley_Conv38hp_20190125.xlsx, this line should read:

$call copy UK-Wheat-OSR-Barley_Conv38hp_20190125.xlsx Orinoquia_Tables.xlsx

Any time the relevant spreadsheet name changes, this line must also be changed so that the correct data is used.

A copy of the GAMS source code for the HFH-LP is in Appendix A. This code has been pasted into an MSWORD file to provide easier access for readers who have not yet installed the GAMS software. Data spreadsheets for the large conventional, smaller conventional, HFH sized conventional equipment and HFH autonomous equipment sets are in Appendices B, C, D and E. The data spreadsheet for HFH scale equipment with human drivers Appendix B is identical to the HFH autonomous equipment data spreadsheet in Appendix E except that a human driver is required whenever a tractor or combine is being used.

Table S1 – HFH LP activities and yields

|  |  |  | Cereal Yield, t/ha | OSR Yield, t/ha |
| --- | --- | --- | --- | --- |
| Winter_Wheat_Oct/OSR_Sept | |  | 9.1 | 3.75 |
| Winter_Wheat_Oct/OSR_Oct | |  | 8.72 | 3.75 |
| Winter_Wheat_Nov/OSR_Sept | | | 7.84 | 3.75 |
| Winter_Wheat_Nov/OSR_Oct_Late | | | 7.84 | 3.24 |
| Spring_Barley_Jan/OSR_Sept | |  | 4.1 | 3.75 |
| Spring_Barley_Jan/OSR_Oct | |  | 3.68 | 3.75 |
| Spring_Barley_Jan/OSR_Oct_Late | | | 3.68 | 3.24 |
| Spring_Barley_Feb/OSR_Sept | |  | 5.54 | 3.75 |
| Spring_Barley_Feb/OSR_Oct | |  | 4.6 | 3.75 |
| Spring_Barley_Feb/OSR_Oct_Late | | | 4.6 | 3.24 |
| Spring_Barley_March/OSR_Sept | | | 6 | 3.75 |
| Spring_Barley_March/OSR_Oct | | | 5.18 | 3.75 |
| Spring_Barley_March/OSR_Oct_Late | | | 5.18 | 3.38 |

| Table S2. Machine inventory and cost for equipment scenarios in the HFH-LP, all monetary amounts in £. | | | | | | | | |  |  |
| --- | --- | --- | --- | --- | --- | --- | --- | --- | --- | --- |
| Machine | Initial Investment | Useful Life | Opportunity cost of capital | Annual Depr. | Insurance as % of investment | Ins. | Repair and Maintenance as % of initial | Repair and Maintenance | Fuel and Lubricant | Annual Cost - Whole Farm |
| Conventional, large | | | | | | | | | | |
| Tractor, about 296 hp | 250000 | 20 | 7500 | 12500 | 0.01 | 2500 | 0.02 | 5000 | 5000 | 32500 |
| Drill, 6 m | 85000 | 10 | 2550 | 8500 | 0.0025 | 212.5 | 0.02 | 1700 | 0 | 12963 |
| Sprayer, trailed 36 m boom | 70000 | 10 | 2100 | 7000 | 0.0025 | 175 | 0.02 | 1400 | 0 | 10675 |
| Twin disc spreader, 24 m, hopper >2500 liter | 6500 | 10 | 195 | 650 | 0.0025 | 16.25 | 0.02 | 130 | 0 | 991 |
| Combine, 5 m header | 250000 | 20 | 7500 | 12500 | 0.01 | 2500 | 0.02 | 5000 | 5000 | 32500 |
| Chaser bin* | 48000 | 20 | 1440 | 2400 | 0.0025 | 120 | 0.02 | 960 | 0 | 4920 |
| Trailer for Hauling to Farmstead | 14000 | 20 | 420 | 700 | 0.0025 | 35 | 0.02 | 280 | 0 | 1435 |
| Total | 723500 |  |  |  |  |  |  |  |  | 95984 |
| Conventional - smallest direct drill equipment in Agro Consultants | | | | | | | | | | |
| Tractor, about 150 hp | 80000 | 20 | 2400 | 4000 | 0.01 | 800 | 0.02 | 1600 | 1600 | 10400 |
| Drill, 3 m | 52000 | 10 | 1560 | 5200 | 0.0025 | 130 | 0.02 | 1040 | 0 | 7930 |
| Sprayer, trailed 24 m boom | 34000 | 10 | 1020 | 3400 | 0.0025 | 85 | 0.02 | 680 | 0 | 5185 |
| Twin disc spreader, 24 m | 6500 | 10 | 195 | 650 | 0.0025 | 16.25 | 0.02 | 130 | 0 | 991 |
| Combine, 4.5 m header | 125000 | 20 | 3750 | 6250 | 0.01 | 1250 | 0.02 | 2500 | 2500 | 16250 |
| Chaser bin* | 48000 | 20 | 1440 | 2400 | 0.0025 | 120 | 0.02 | 960 | 0 | 4920 |
| Trailer for Hauling to Farmstead | 14000 | 20 | 420 | 700 | 0.0025 | 35 | 0.02 | 280 | 0 | 1435 |
| Total | 359500 |  |  |  |  |  |  |  |  | 47111 |

| Table S1. Machine inventory and cost for equipment scenarios in the HFH-LP, all monetary amounts in £ (Continued) | | | | | | | | | | |
| --- | --- | --- | --- | --- | --- | --- | --- | --- | --- | --- |
| Machine | Initial Investment | Useful Life | Opportunity Cost of Capital | Annual Depr. | Insurance as % of investment | Ins. | Repair and Maintenance as % of initial | Repair and Maintenance | Fuel and Lubricant | Annual Cost – Whole Farm |
| Autonomous - HFH example* |  |  |  |  |  |  |  |  |  |  |
| Tractor, 38 hp, Hydrostatic | 15000 | 20 | 450 | 750 | 0.01 | 150 | 0.02 | 300 | 300 | 1950 |
| Drill | 12000 | 10 | 360 | 1200 | 0.0025 | 30 | 0.02 | 240 | 0 | 1830 |
| Sprayer, trailed 4 m boom | 4900 | 10 | 147 | 490 | 0.0025 | 12.25 | 0.02 | 98 | 0 | 747 |
| Twin disc spreader, 12 m | 3500 | 10 | 105 | 350 | 0.0025 | 8.75 | 0.02 | 70 | 0 | 534 |
| Combine, 2 m head | 28000 | 20 | 840 | 1400 | 0.01 | 280 | 0.02 | 560 | 560 | 3640 |
| Trailer for Hauling to Farmstead | 4500 | 20 | 135 | 225 | 0.0025 | 11.25 | 0.02 | 90 | 0 | 461 |
| RTK GPS and autopilot | 23262 | 10 | 697.86 | 2326.2 | 0.0025 | 58.16 | 0.02 | 465.24 | 0 | 3547 |
| Total | 91162 |  |  |  |  |  |  |  |  | 11547 |
| * The calculations here are for the HFH autonomous equipment. For the HFH farm sized equipment operated by a human, omit the RTK, GPS and autopilot. HFH autonomous equipment retains a seat and steering wheel for a human operator if needed (e.g. to move the equipment on a public road from farm to farm). | | | | | | | | | | |

Table S3 – Hardware and software needed to retrofit the HFH equipment for autonomous operation.

| Type of Equipment | Item | Cost |  |  |
| --- | --- | --- | --- | --- |
| Safety Equipment |  |  |  |  |
|  | Laser | £3,282.00 |  |  |
|  | Remote Emergency Stop | £ 75.00 |  |  |
|  | Stop Button System | £ 65.00 |  |  |
| Control System |  |  |  |  |
|  | GPS | £2,300.00 |  |  |
|  | Autopilot | £ 112.00 |  |  |
| Control Adaptations |  |  |  |  |
|  | Steering motor | £ 768.00 |  |  |
|  | Drive control | £ 860.00 |  |  |
|  | Linkage control | £ 430.00 |  |  |
| Camera Feedback | CCTC Cams | £ 340.00 |  |  |
| Communications |  |  |  |  |
|  | WIFI | £ 100.00 |  |  |
|  | RC system | £ 413.00 |  |  |
| Consumables | Boxes/connectors Etc. | £ 600.00 |  |  |
|  | Total | £9,345.00 |  |  |
|  |  |  |  |  |
| * Combine has extra: Laser £3282 and 3 actuators + drivers £1290 (3x£430). | | | | |

| Table S4. Estimate hectares per hour and hours per hectare for key items in each equipment set | | | | | | |
| --- | --- | --- | --- | --- | --- | --- |
| Machine | width | overlap | Field speed, km/hr | Field Efficiency* | Area/hr** | hr/ha |
| HFH equipment set: | |  |  |  |  |  |
| Drill | 1.5 | 10% | 3.25 | 70% | 0.31 | 3.26 |
| Sprayer | 7 | 10% | 5 | 70% | 2.21 | 0.45 |
| Combine | 2 | 10% | 3.25 | 70% | 0.41 | 2.44 |
| Larger Conventional: | | |  |  |  |  |
| Drill | 6 | 10% | 5 | 70% | 1.89 | 0.53 |
| Sprayer | 36 | 10% | 10 | 70% | 22.68 | 0.04 |
| Combine | 7.5 | 10% | 3 | 70% | 1.42 | 0.71 |
| Smaller Conventional: | | |  |  |  |  |
| Drill | 3 | 10% | 5 | 70% | 0.95 | 1.06 |
| Sprayer | 24 | 10% | 10 | 70% | 15.12 | 0.07 |
| Combine | 4.5 | 10% | 3 | 70% | 0.85 | 1.18 |
|  |  |  |  |  |  |  |
| * Allowing for turning, field shape etc. See Table 3.3 p. 103, Witney | | | | | |  |
| ** Witney, p. 98 | |  |  |  |  |  |
| *** Assumes all drilling in September and October for Wheat and OSR. Barley drilling has a bigger window and is unlikely to be constraining. | | | | | | |
| All harvest is in July and August | | | | | | |

| Table S5 - Field Operations and Equipment Time per Hectare by Crop, Month and Equipment Set for Optimum Yields, days/ha | | | | | | | | | | |  |  |
| --- | --- | --- | --- | --- | --- | --- | --- | --- | --- | --- | --- | --- |
|  | Jan | Feb | Mar | Apr | May | Jun | Jul | Aug | Sep | Oct | Nov | Dec |
| Winter Wheat Field Operations |  |  | Top dressing & spraying |  | Top dressing & spraying | Spraying |  | Harvest |  | Predrill herbicide & Drill |  |  |
| Larger conventional |  |  | 0.0110 |  | 0.0110 | 0.0055 |  | 0.1761 |  | 0.0716 |  |  |
| Smaller conventional |  |  | 0.0165 |  | 0.0165 | 0.0083 |  | 0.2941 |  | 0.1405 |  |  |
| HFH set |  |  | 0.1984 |  | 0.1984 | 0.0992 |  | 0.6098 |  | 0.5062 |  |  |
| OSR Field Operations |  | N topdress | Fungal Spray | Insect Spray |  | Late insect or fungal spray | Harvest |  | Predrill herbicide & Drill |  |  |  |
| Larger conventional |  | 0.0055 | 0.0055 | 0.0055 |  | 0.0055 | 0.1761 |  | 0.0716 |  |  |  |
| Smaller conventional |  | 0.0083 | 0.0083 | 0.0083 |  | 0.0083 | 0.2941 |  | 0.1405 |  |  |  |
| HFH set |  | 0.0992 | 0.0992 | 0.0992 |  | 0.0992 | 0.6098 |  | 0.5062 |  |  |  |
| Spring Barley Field Operations | Predrill herbicide & Drill |  |  | Top dressing & spraying | Top dressing & spraying |  |  | Desiccant &Harvest |  |  |  |  |
| Larger conventional | 0.0716 |  |  | 0.0110 | 0.0110 |  |  | 0.1816 |  |  |  |  |
| Smaller conventional | 0.1405 |  |  | 0.0165 | 0.0165 |  |  | 0.3024 |  |  |  |  |

References for Supplementary Materials:

Agro Business Consultants (2018). *The Agricultural Budgeting & Costing Book No. 87.* Melton Mowbray, Leicestershire, UK: Agro Business Consultants Ltd.

Dobbins, C.L., Han, Y., Preckel, P.V., and Doster, D.H. (1994).  *Purdue Crop/Livestock Linear Program (PC/LP) Version 3.2.* West Lafayette, IN, USA: Cooperative Extension Service, Purdue University.

Dobbins, C.L., Preckel, P.V., Han, Y., Doster D.H. and Horan, B. (1990). A Decision Support System for Alternative Cropping Systems. *Proceedings of the Third International Conference on Computers in Agricultural Extension Programs*. Orlando, Florida, 282-287.

Dobbins, C.L., Preckel, P.V., Han, Y., and Doster D.H. (1992). An Application of Linear Programming to Planning Crop Systems. *Proceedings of the Fourth International Conference on Computers in Agricultural Extension Programs*. Orlando, Florida, 376-381.

Finch, H., Samuel, A. and Lane, G. (2014). *Lockhart & Wiseman’s Crop Husbandry Including Grassland.* Cambridge, UK: Woodhead Publishing Series in Food Science, Technology and Nutrition, Number 277.

Fontanilla, C. (2019). *Parameter Editing for the Orinoquia Agricultural Linear Programming Model* Colombia Purdue Partnership, Purdue University. [https://www.purdue.edu/colombia/partnerships/orinoquia/docs/ParameterEd.pdf](about:blank).

Accessed 7 August 2020

Griffin, T. Lambert, D., Lowenberg-DeBoer, J., (2005). Economics of GPS lightbar navigation and auto-guidance technologies. *Precision Agriculture-2005*, John Stafford, editor, 581-587, The Netherlands: Wageningen Academic Publishers.

Lowenberg-DeBoer, J. (1999). GPS based guidance systems for agriculture. *Purdue Agricultural Economics Report.* Purdue University, December, 8-9. <https://ag.purdue.edu/agecon/Documents/PAER_December%201999.pdf>. Assessed 7 August 2020.

Ortiz, B., Balkcom, K., Duzy, L., Van Santen, E. Hartzog, D. (2013). Evaluation of agronomic and economic benefits of using RTK-GPS-based auto-steer guidance systems for peanut digging operations. *Precision Agriculture,* **14**: 357-375.

Outsider’s Guide (1999). *The Outsider’s Guide: The Book for Anyone Ploughing into Agriculture at any Time.* Phil and Sally Cottle, eds., Rylands, Stow, Lincoln, UK.

Preckel, P., Han, Y., Dobbins, C., Doster, D. (1992). *Purdue Crop/Livestock Linear Program Formulation.* Purdue University Agricultural Experiment Station Bulletin No. 634.

Preckel, P., Fontanilla, C., Lowenberg-DeBoer, J. and Sanders, J. (2019). *Orinoquia Agricultural Linear Programming Model – Documentation.* Colombia Purdue Partnership, Purdue University. [https://www.purdue.edu/colombia/partnerships/orinoquia/docs/OrinoquiaLPDoc.pdf](about:blank). Accessed 7 August 2020.

United Kingdom Department of Environment, Food and Rural Affairs (DEFRA) (2018a). *England Regional Profiles.* Kings Pool, York, UK.

United Kingdom Department of Environment, Food and Rural Affairs (2018b). *Farm type breakdown for commercial holdings*.
<https://www.gov.uk/government/statistical-data-sets/structure-of-the-agricultural-industry-in-england-and-the-uk-at-june>*.* Accessed 7 August 2020.

Witney, B. (1988). *Choosing and Using Farm Machines.* Edinburgh Scotland, UK: Longman Scientific & Technical.
